# Correlates of HIV testing and receipt of test results in addiction health services in Los Angeles County

**DOI:** 10.1186/s13011-015-0026-1

**Published:** 2015-08-07

**Authors:** Jemima A. Frimpong, Erick G. Guerrero, Yinfei Kong, Gary Tsai

**Affiliations:** Department of Health Policy and Management, Mailman School of Public Health, Columbia University, 722 West 168th Street, New York, NY 10032 USA; School of Social Work, University of Southern California, 655 West 34th Street, Los Angeles, CA 90089 USA; Los Angeles County Department of Public Health, Substance Abuse Prevention and Control, 1000 South Fremont Avenue, Building A-9 East, Alhambra, CA 91803 USA

**Keywords:** HIV testing, Receipt of HIV test results, Addiction health services, Substance use disorder treatment

## Abstract

**Background:**

HIV testing and receipt of HIV test results among individuals with substance use disorders is less than optimal. We examined rates and correlates of HIV testing and receipt of test results in one of the largest public addiction health services systems in the United States.

**Methods:**

The study included 139,516 adult clients in treatment between 2006 and 2011. We used logistic regression models to examine associations between predisposing, enabling, and need factors and two dependent variables, HIV testing rates and receipt of test results. Associations were considered statistically significance at *p* < .01.

**Results:**

We found that 64 % of clients reported being tested for HIV, of whom 85 % reported receiving their test results. Likelihood of being tested was positively associated with being female, a minority, homeless, employed, having prior treatment episodes, comorbidities, injection drug use, or a history of mental illness. It was negatively associated with alcohol or marijuana as primary drug. Receipt of test results was more likely among clients on medication (methadone or buprenorphine) or whose method of drug use was smoking, inhalation, or injecting; it was less likely among older clients and those with more outpatient psychiatric visits.

**Conclusions:**

Findings from this study may inform strategies and targeting of population groups to improve HIV testing practices and ultimately increase awareness of infection status among clients of addiction health services.

## Introduction

More than 1.2 million people in the United States are living with HIV, with an estimated incidence of 50,000 infections each year [1]. HIV prevalence among individuals with substance use disorders, particularly persons who inject drugs (PWID), is high compared to the general population [2, 3]. Recent data showed that only 49 % of PWID reported ever been tested [4]. For individuals infected with HIV to benefit from antiretroviral therapy, they must be tested and diagnosed, receive results of their HIV status, be linked to HIV medical care, receive treatment, remain continuously engaged in HIV care, and adhere to treatment [5–11]. HIV testing is necessary to set this HIV care cascade in motion and address the HIV burden, but testing alone is not sufficient. Clients tested for HIV must also be made aware or informed of their infection status. The Centers for Disease Control and Prevention (CDC) has estimated that nearly 16 % of HIV positive individuals in the United States are unaware of their infection status [12]. Knowledge of one’s infection status may lead to reductions in risky behaviors associated with transmission of the virus. Indeed, individuals who test negative and receive posttest counseling may also adopt risk-reduction behaviors that may prevent HIV infection.

Although individuals with substance use disorders are at higher risk of HIV infection, the availability of testing and receipt of test results is less than optimal [13, 14]. The CDC therefore recommends routine HIV testing for risk groups, including individuals with substance use disorders [15]. Yet, less than half of substance use disorders treatment programs in the nation offer HIV testing to their clients [16, 17]. Few studies have examined multicomponent factors associated with testing and receipt of test results; particularly factors associated with characteristics of clients and substance use disorder treatment programs [14]. The purpose of the current study was to examine the rates and correlates of HIV testing and receipt of HIV test results among clients receiving treatment services in one of the largest publicly funded addiction treatment systems in the United States (Los Angeles County, California).

The Gelberg-Anderson Behavioral Model for Vulnerable Populations informed our conceptualization of this study [18]. The model is an extension of the Anderson Behavioral Model, which describes the relationships among predisposing, enabling, and need factors that explain health services use in the general population. Predisposing factors include demographic and other personal characteristics and health care social structures that influence the likelihood of obtaining care. These factors are often present before illness (e.g., gender, age, education). Enabling factors refer to personal, family, and community resources that support or are influential to obtaining health services. Enabling factors serve as facilitators of health services use and also include employment status, number of medical visits, and insurance status. Need factors generally refer to the need for health services, whether actual or perceived, and conditions of special relevance to the population of interest that motivate individuals to seek needed health care services [19–23]. Clinical evaluation of patients and personal practices may also affect an individual’s vulnerability status and are considered a measure of need. Specifically, factors such as injection drug use, mental illness, or hepatitis C virus (HCV) infections often serve as the motivation or reason for obtaining health services. The Gelberg-Anderson model has been extensively applied to HIV testing and receipt of test results among individuals who are homeless or have serious mental illness [24–26]. However, there is limited empirical information about the predisposing, enabling, and need factors that may explain HIV testing and receipt of test results in publicly funded addiction treatment.

Using the Gelberg-Anderson model and findings from prior research on HIV testing practices; we examined the relationship between client and program factors and the receipt of HIV-related services (i.e., rates and predictors of HIV testing and receipt of test results). We expected that predisposing factors (e.g., homelessness) would be negatively correlated with HIV testing and receipt of test results because of clients’ main priorities of food and shelter, as well as inconsistent delivery of care to homeless individuals documented in the literature [27]. We expected that enabling factors (e.g., prior treatment episodes) would be positively associated with HIV testing and receipt of test results. For example, because of greater exposure to the health care system, clients may be more adept at navigating the system and have increased opportunities for HIV testing and receipt of results. Finally, we expected that need factors (e.g., HCV diagnosis, injection drug use) would be positively associated with HIV testing and receipt of test results. These clients may have had experiences that positively influenced their awareness of risk factors for HIV and would thus be more motivated to obtain health services.

Hypothesis 1: Predisposing factors, such as homelessness, would be associated with lower odds of (a) HIV testing and (b) receipt of HIV test results.

Hypothesis 2: Enabling factors, such as prior treatment episodes, would be associated with higher odds of (a) HIV testing and (b) receipt of HIV test results.

Hypothesis 3: Need factors, such as HCV diagnosis, would be associated with higher odds of (a) HIV testing and (b) receipt of HIV test results.

## Methods

### Sampling frame and data collection

This study analyzed a subset of data from 2006 to 2011 from the Los Angeles County Participant Reporting System (LACPRS). This database includes data from all publicly funded substance abuse treatment programs in the most populous county in the United States [28]. This ongoing systemwide evaluation database captures the treatment experience and immediate outcomes of low-income racially and ethnically diverse clients. Of the 141 items in the LACPRS, more than half are standardized scales and questions related to admission, discharge, and health derived from state (California Outcome Measure System) and federal (Treatment Episode Data Set) measurement systems.

Client data in LACPRS are collected during personal interviews at intake and discharge for most individuals. Through the use of standardized instruments, counselors collect information on five major domains: employment status, legal status, substance use profile, substance use history, and medical and psychological status. The collection form includes 10 items from the Addiction Severity Index [29] and the Drug Abuse Reporting Program [30, 31]. These scales have been shown to be reliable measures of substance abuse severity [32], particularly among diverse populations [33], allowing for assessment of client reports from intake to discharge.

Client data used in this study represented 139,516 treatment episodes collected from July 1, 2006, to December 30, 2011. We limited the analysis to outpatient programs because they represent the most common treatment option in Los Angeles County, accounting for more than 70 % of all admissions [34]. Only clients who were admitted and discharged within the same year were included to obtain accurate estimates, due to data coding issues with clients who stayed beyond one calendar year.

The University of Southern California Institutional Review Board reviewed and approved the study.

### Dependent variables

We examined two dependent variables related to HIV services received on- or off-site. Clients were asked at admission and discharge if they had been tested for HIV/AIDS on- or off-site and, if so, whether they had obtained the corresponding results at admission or discharge. We defined two dichotomous dependent variables based on these questions. For HIV testing we used two items: (a) tested for HIV/AIDS on- or off-site at admission (prior to entering treatment) and (b) tested for HIV/AIDS on- or off-site at discharge (while in treatment). For obtaining results, we relied on one item asked of those tested for HIV/AIDS: (a) received result of HIV/AIDS test, reported at admission or discharge. Note that respondents reported being tested for HIV/AIDS prior to and during treatment, but the data did not allow us to differentiate whether testing was provided on- or off-site. We considered all potential outcomes: testing and receipt of results at admission only, discharge only, both, or neither. We did not find differences between individuals tested or individuals who received test results at admission versus discharge (*p* > .05).

### Independent variables

#### Predisposing variables

The following demographic and social structure characteristics were included in our models: sex, age, race and ethnicity, education, and homelessness, measured by self-reports regarding current housing situation. We also included source of referral to an addiction health services program; number of days spent in jail or prison during the previous 30 days (criminal activity); and participation in any social support recovery activities, such as 12-step meetings or interaction with family members or friends supportive of recovery, during the previous 30 days (recovery support). Categories of independent variables, including enabling and need factors, are presented in Table 1.

**Table 1:** Factors associated with HIV testing and receipt of test results among clients in addiction health services

	Full sample	Tested for HIV			Received HIV test results		
	*N* = 139,516	*n* = 89,383			*n* = 76,392		
	*M* (*SD*) or %	*M* (*SD*) or %	*M* Diff^a^	*p* ^b^	*M* (*SD*) or %	*M* Diff^c^	*p* ^b^
*Predisposing factors*							
Age	36.13 (12.51)	36.81 (11.51)	2.18	< .001	36.78 (11.46)	1.73	< .001
Days incarcerated	1.99 (6.61)	2.28 (7.07)	0.71	< .001	2.29 (7.09)	0.55	< .001
Recovery support	3.73 (8.24)	4.78 (9.13)	2.76	< .001	4.99 (9.31)	2.70	< .001
Gender							
Female	0.35	0.39		< .001	0.39		< .001
Male	0.64	0.61			0.61		
Race							
White	0.34	0.36		< .001	0.36		< .001
Black	0.23	0.23		.047	0.23		< .001
Latino	0.38	0.37		< .001	0.36		< .001
Asian	0.05	0.05		< .001	0.05		< .001
Education							
Less than high school	0.08	0.06		< .001	0.06		< .001
High school	0.75	0.74		< .001	0.74		< .001
College or higher	0.17	0.20		< .001	0.20		< .001
Homeless	0.24	0.29		< .001	0.28		< .001
Referral							
Self	0.39	0.39		.072	0.39		.001
Community	0.13	0.11		< .001	0.11		< .001
Proposition 36	0.21	0.21		< .001	0.22		< .001
Drug court	0.08	0.08		< .001	0.09		< .001
Social services	0.19	0.20		< .001	0.20		< .001
*Enabling factors*							
Days on waiting list	3.07 (9.26)	3.42 (8.95)	0.75	< .001	3.21 (8.58)	0.11	.043
Prior treatment episodes	1.66 (3.58)	1.95 (3.70)	0.84	< .001	1.97 (3.62)	0.73	< .001
Outpatient medical visits	0.15 (1.58)	0.18 (1.74)	0.07	< .001	0.17 (1.66)	0.04	< .001
Outpatient psychiatric visits	0.22 (2.00)	0.24 (2.05)	0.04	< .001	0.24 (2.03)	0.02	.092
Treatment type							
Outpatient	0.49	0.42		< .001	0.43		< .001
Methadone	0.04	0.02		< .001	0.03		< .001
Residential	0.46	0.55		< .001	0.54		< .001
Employed at admission	0.12	0.12		.212	0.12		< .001
Medication							
None	0.81	0.79		< .001	0.80		< .001
Methadone/LAAM	0.06	0.05		< .001	0.06		.429
Buprenorphine	0.64	0.01		.927	0.01		.179
Other	0.11	0.15		< .001	0.13		< .001
*Need factors*							
Days of primary drug use	11.19 (12.49)	11.65 (12.65)	1.18	< .001	11.30 (12.52)	0.41	< .001
Days of injection drug use	3.35 (9.11)	3.65 (9.37)	1.28	< .001	3.39 (9.05)	0.48	< .001
Primary drug							
Heroin	0.17	0.19		< .001	0.18		< .001
Alcohol	0.22	0.21		< .001	0.21		< .001
Methamphetamine	0.25	0.27		< .001	0.28		< .001
Cocaine or crack	0.16	0.17		< .001	0.18		< .001
Marijuana or hashish	0.14	0.10		< .001	0.10		< .001
Other	0.05	0.06		< .001	0.06		< .001
Method of drug use							
Oral	0.27	0.26		< .001	0.26		< .001
Smoking	0.51	0.50		< .001	0.51		< .001
Inhalation	0.06	0.06		< .001	0.06		< .001
Injection	0.16	0.18		< .001	0.17		< .001
Other	0.07	0.01		.058	0.01		.781
HCV diagnosis							
Yes	0.06	0.09		< .001	0.08		< .001
No	0.94	0.91			0.92		
STD diagnosis							
Yes	0.04	0.06		< .001	0.06		< .001
No	0.96	0.94			0.94		
History of mental illness							
Yes	0.24	0.27		< .001	0.28		< .001
No	0.76	0.73			0.72		

*HCV* hepatitis C virus, *LAAM* levo-α-acetylmethadol, *STD* sexually transmitted disease

^a^Mean difference between clients tested for HIV and clients not tested for HIV

^b^Chi-square test for categorical variables and *t*-tests for continuous variables

^c^Mean difference between clients who received HIV test results and clients who did not receive HIV test results

#### Enabling variables

We included clients’ employment status at admission, number of days on a waiting list before being admitted to the treatment program, and treatment type (i.e., outpatient, methadone, or residential treatment). Studies have shown that type of treatment program is associated with the availability of preventive services such as HIV and HCV testing [13, 35]. We also included variables indicating whether a patient had prior treatment episodes, received medication for substance abuse, received outpatient and inpatient health services, and received substance abuse treatment, medical, and psychiatric services.

#### Need variables

Need factors of special relevance to substance abuse populations included in the analysis were clients’ primary drug of choice, number of days of primary drug use during the previous 30 days, and primary method of drug use [36]. The frequency of injection drug use is associated with transmission of HIV [37, 38]. Hence, we included a variable measuring the number of days during which a client injected drugs during the previous 30 days. Last, whether a client had been diagnosed with a sexually transmitted disease or HCV was included because it related to modes of transmission and coinfection rates [39–42].

### Data analysis

We used univariate statistics to describe client characteristics and summarize rates of HIV testing and receipt of HIV test results. Chi-square tests were performed for categorical variables and *t*-tests for continuous variables. We then conducted parallel bivariate and multivariate logistic regressions, which corresponded to the two dependent variables. Bivariate analysis examined associations between predisposing, enabling, and need factors and dependent variables. Multiple random effect logistic regression was conducted to identify independent predictors of HIV testing and receipt of test results. We used a backward elimination strategy to determine the most parsimonious set of variables significant at *p* < .05. These variables were included in the final models. Due to relative large sample size, we focused our interpretation of results on factors associated with HIV testing or receipt of HIV test results at the statistical significance level of *p* < .01 and present 99 % confidence intervals. Results are presented as odds ratios (*OR*), with which the relationship between exposure (independent variable) and an outcome (e.g., HIV testing) are compared to no exposure. When this relationship is positive, we reported higher odds (*OR* > 1) and lower odds (*OR* < 1) when the relationship is negative. To account for the hierarchical structure of the data, which refers to the nesting of clients in programs, we used the CLUSTER option in the main function LOGIT of Stata. All analyses were performed using Stata/SE Version 12.

## Results

### Full sample

Approximately 139,516 clients of addiction health services programs in Los Angeles County were included in the analysis. Nearly 28 % of clients reported more than one treatment episode, with an average of 1.8 treatment episodes for the full sample. More than half of the total final sample reported being tested for HIV during their time in treatment (64 %). Among those tested, 85 % reported that they received their test results. About 65 % of the full sample was male, with Blacks and Latinos accounting for 60 %. Nearly 24 % self-reported as being homeless and the majority of clients were self-referred to treatment (39 %). Most of the clients in the sample were not receiving pharmacotherapy (81 %). Methamphetamine (25 %), followed by alcohol (22 %), was the primary drug of choice in the study sample. Approximately 16 % of clients injected drugs. HCV and sexually transmitted disease (STD) diagnosis in the sample was 6 % and 4 %, respectively, and 24 % reported a history of mental illness.

### Tested for HIV

#### Predisposing factors

Compared to the full sample, the subsample that reported HIV testing had a higher proportion of women, Whites, college-educated individuals, homeless individuals, and those referred by social services (see Table 1, bivariate analysis). Multivariate analysis (Table 2) showed no support for Hypothesis 1a, i.e., predisposing factors were not associated with lower odds of HIV testing. The odds of HIV testing were higher among women than men (*OR* = 1.527, 99 % CI = 1.277, 1.826) and among Blacks and Latinos compared to Whites (*OR* = 1.299, 99 % CI = 1.039, 1.623 and *OR* = 1.126, 99 % CI = 0.987, 1.285, respectively). Compared to clients without a college education, those with a college education reported higher odds of HIV testing (*OR* = 1.991, 99 % CI = 1.613, 2.457) after adjusting for other explanatory variables. Additionally, the odds of HIV testing were higher among clients who were homeless (*OR* = 1.414, 99 % CI = 1.161, 1.724); received a referral from Proposition 36 (court referral to treatment instead of incarceration; *OR* = 1.388, 99 % CI = 1.032, 1.866) or social services (*OR* = 1.282, CI = 0.961, 1.710); and had recovery support (*OR* = 1.039, 99 % CI = 1.029, 1.049).

**Table 2:** Logistic regression on HIV testing and receipt of test results among clients in addiction health services

Independent variables	HIV Testing			Receipt of HIV test results		
	*n* = 89,383			*n* = 76,392		
	*OR*	*SE*	99 % CI	*OR*	*SE*	99 % CI
*Predisposing factors*						
Female	1.527**	0.106	1.277, 1.826	1.454**	0.123	1.169, 1.809
Age	0.998	0.001	0.994, 1.002	0.997	0.001	0.994, 1.000
Race^a^						
Black	1.299*	0.112	1.039, 1.623	1.183	0.099	0.952, 1.468
Latino	1.126	0.058	0.987, 1.285	1.003	0.049	0.884, 1.138
Asian	0.945	0.052	0.820, 1.089	0.911	0.058	0.773, 1.074
Education^b^						
High school	1.434**	0.121	1.154, 1.782	1.336**	0.105	1.090, 1.636
College or higher	1.991**	0.163	1.613, 2.457	1.930**	0.141	1.598, 2.331
Homeless	1.414**	0.109	1.161, 1.724	1.158	0.122	0.882, 1.519
Referral^c^						
Community	1.146	0.144	0.829, 1.584	1.225	0.168	0.860, 1.746
Proposition 36	1.388*	0.160	1.032, 1.866	1.354	0.169	0.980, 1.869
Drug court	1.225	0.217	0.777, 1.933	1.307	0.238	0.818, 2.089
Social services	1.282	0.143	0.961, 1.710	1.215	0.232	0.743, 1.987
Days incarcerated	1.010	0.006	0.995, 1.024	1.005	0.005	0.992, 1.019
Recovery support	1.039**	0.004	1.029, 1.049	1.034**	0.004	1.023, 1.045
*Enabling factors*						
Employed at admission	1.174*	0.070	1.006, 1.370	1.241**	0.079	1.055, 1.461
Days on waiting list	0.998	0.007	0.981, 1.015	0.993	0.005	0.979, 1.006
Treatment type^d^						
Methadone	0.478	0.272	0.111, 2.069	0.612	0.344	0.144, 2.601
Residential	1.738**	0.263	1.177, 2.565	1.672*	0.306	1.044, 2.680
Prior treatment episodes	1.069**	0.019	1.022, 1.118	1.048**	0.014	1.013, 1.083
Medication						
None						
Methadone/LAAM	1.144	0.150	0.817, 1.603	1.754**	0.302	1.126, 2.733
Buprenorphine	1.220	0.113	0.962, 1.548	1.428	0.226	0.949, 2.146
Other	1.598*	0.291	1.000, 2.554	1.288	0.434	0.541, 3.069
Outpatient medical visits	1.000	0.005	0.986, 1.014	0.994	0.008	0.972, 1.015
Outpatient psychiatric visits	0.992	0.008	0.973, 1.012	0.981	0.009	0.959, 1.004
*Need factors*						
Primary drug^e^						
Alcohol	0.783	0.081	0.600, 1.020	1.018	0.119	0.754, 1.375
Methamphetamine	1.023	0.072	0.853, 1.226	1.189	0.127	0.902, 1.566
Cocaine or crack	0.889	0.068	0.730, 1.083	1.069	0.116	0.808, 1.413
Marijuana or hashish	0.753**	0.051	0.632, 0.897	0.897	0.090	0.693, 1.163
Other	0.961	0.078	0.780, 1.184	1.238	0.128	0.948, 1.617
Days of primary drug use	1.003	0.003	0.996, 1.010	1.002	0.003	0.993, 1.011
Method of drug use^f^						
Smoking	1.080	0.079	0.894, 1.306	1.165	0.073	0.992, 1.368
Inhalation	1.217	0.101	0.982, 1.509	1.225*	0.089	1.016, 1.476
Injection	1.389**	0.128	1.095, 1.762	1.602**	0.155	1.248, 2.057
Other	1.285	0.260	0.763, 2.164	1.133	0.235	0.664, 1.932
Days of injection drug use	1.003	0.005	0.992, 1.015	0.991	0.006	0.975, 1.007
HCV diagnosis	2.346**	0.367	1.568, 3.510	1.632**	0.206	1.180, 2.258
STD diagnosis	3.403**	0.442	2.435, 4.756	2.535**	0.333	1.807, 3.555
History of mental illness	1.487**	0.121	1.206, 1.834	1.518**	0.140	1.198, 1.925

*CI* confidence interval, *HCV* hepatitis C virus, *LAAM* levo-α-acetylmethadol, *STD* sexually transmitted disease

^a^White was reference group

^b^Less than high school was reference group

^c^Self-referral was reference group

^d^Outpatient treatment was reference group

^e^Heroin was reference group

^f^Oral was reference group

*p < .01. **p < .001

#### Enabling factors

The subsample that reported HIV testing also reported more days on a waiting list, more treatment episodes, and more outpatient medical and psychiatric visits. This subsample also reported a higher proportion of clients in residential treatment, a lower proportion of outpatient and methadone treatment, and less use of methadone and other medications (see Table 1).

Hypothesis 2a was supported, i.e., enabling factors were associated with higher odds of HIV testing (see Table 2). The odds of receiving HIV testing were higher among clients who were employed at admission (*OR* = 1.174, 99 % CI = 1.006, 1.370) and had prior treatment episodes (*OR* = 1.069, 99 % CI = 1.022, 1.118) after adjusting for other explanatory variables. In addition, clients in residential treatment had higher odds of receiving HIV testing compared to other treatment types (*OR* = 1.738, 99 % CI = 1.177, 2.565). Last, clients receiving buprenorphine medication had higher odds of receiving HIV testing when compared to other medication use (*OR* = 1.220, 99 % CI = 0.962, 1.548).

#### Need factors

Individuals who reported HIV testing had higher proportions of heroin, methamphetamine, and cocaine use and a higher rate of injection as the method of drug use. This sample reported less use of alcohol and marijuana. This sample also reported higher rates of HCV, STD, and history of mental illness (see Table 1).

Partial support was found for Hypothesis 3a, i.e., some need factors were associated with higher odds of HIV (see Table 2). The odds of HIV testing among clients whose primary drug was alcohol or marijuana were significantly lower relative to heroin (*OR* = 0.783, 99 % CI = 0.601, 1.021 and *OR* = 0.753, 99 % CI = 0.632, 0.897, respectively). Inhalation (*OR* = 1.217, 99 % CI = 0.982, 1.509) and injection (*OR* = 1.389, 99 % CI = 1.095, 1.762) as methods of drug use were associated with higher odds of receiving HIV testing relative to oral drug use. Last, the odds of HIV testing were higher for individuals with a history of mental illness (*OR* = 1.487, 99 % CI = 1.206, 1.834), more than twice as high for individuals with a HCV diagnosis (*OR* = 2.346, 99 % CI = 1.568, 3.510), and more than 3 times as high for individuals with a STD diagnosis (*OR* = 3.403, 99 % CI = 2.435, 4.756), relative to their counterparts with no history of mental illness, no HCV diagnosis, and no STD diagnosis, respectively.

### Receipt of HIV test results

#### Predisposing factors

Clients reporting receipt of HIV test results had a higher proportion of women, Whites, college-educated individuals, homeless individuals, and clients referred by social services (see Table 1).

Hypothesis 1b was partially supported, i.e., some predisposing factors were associated with lower odds of receipt of HIV test results, as presented in Table 2. The odds of receipt of HIV test results decreased with age (*OR* = 0.997, 99 % CI = 0.994, 1.000). However, odds of receiving HIV test results were higher among women (*OR* = 1.454, 99 % CI = 1.169, 1.809), Blacks (*OR* = 1.183, 99 % CI = 0.952, 1.468), college-educated individuals (*OR* = 1.930, 99 % CI = 1.598, 2.331), and clients who had recovery support (*OR* = 1.034, 99 % CI = 1.023, 1.045). Last, the odds of receipt of HIV test results were higher for clients who received a Proposition 36 referral compared to other referral sources (*OR* = 1.354, 99 % CI = 1.023, 1.450).

#### Enabling factors

Clients who received HIV test results also reported more days on a waiting list, more treatment episodes, more outpatient medical and psychiatric visits, an increased likelihood of being in residential treatment, and a lower likelihood of receiving outpatient and methadone treatment. This sample also reported less use of methadone and other medications (see Table 1).

Partial support was found for Hypothesis 2b, i.e., some predisposing factors were associated with higher odds of receipt of HIV test results (see Table 2). The odds of receipt of HIV test results were higher among clients who were employed at admission (*OR* = 1.241, 99 % CI = 1.055, 1.461) and had prior treatment episodes (*OR* = 1.048, 99 % CI = 1.013, 1.083). However, more outpatient psychiatric visits resulted in lower odds of receipt of HIV test results (*OR* = 0.981, CI = 0.959, 1.004). In addition, clients in residential treatment had higher odds of receiving HIV test results compared to other treatment types (*OR* = 1.672, 99 % CI = 1.044, 2.680). Last, clients using methadone and buprenorphine medication had higher odds of receiving HIV test results when compared to other medications (*OR* = 1.754, 99 % CI = 1.126, 2.733 and *OR* = 1.428, 99 % CI = 0.949, 2.146, respectively).

#### Need factors

Relative to the full sample, individuals who received HIV test results reported higher proportions of heroin, methamphetamine, and cocaine use and a higher rate of injection as the method of drug use. This sample reported less use of alcohol and marijuana. This sample also reported higher rates of HCV, STD, and history of mental illness (see Table 1).

Partial support was found for Hypothesis 3b, i.e., some need factors were associated with higher odds of receipt of HIV test results (see Table 2). Smoking (*OR* = 1.165, 99 % CI = 0.992, 1.368), inhalation (*OR* = 1.225, 99 % CI = 1.016, 1.476), and injection (*OR* = 1.602, 99 % CI = 1.248, 2.057) as methods of drug use resulted in higher odds of receiving HIV test results. Last, the odds of receipt of HIV test results were higher among clients with a history of mental illness (*OR* = 1.518, 99 % CI = 1.198, 1.925), HCV diagnosis (*OR* = 1.632, 99 % CI = 1.180, 2.258), and STD diagnosis (*OR* = 2.535, 99 % CI = 1.807, 3.555).

## Discussion

Using data from Los Angeles County, we found that despite federal efforts by regulatory agencies such as the CDC to increase HIV testing and receipt of test results among high-risk populations, only 64 % of clients of one of the largest addiction health services programs reported being tested for HIV. Among those tested, however, 85 % reported receiving their test results. These results illustrate the need for programs that further emphasize testing and awareness of HIV status among clients of addiction health services. The high proportion of clients receiving test results suggests that there may be client and program factors facilitating awareness of HIV status.

Guided by the Gelberg-Anderson model, we tested the extent to which predisposing, enabling, and need factors were associated with HIV testing and receipt of HIV test results. Except for Hypothesis 1a, findings lent partial support to all hypotheses tested. Overall, predisposing factors were not associated with lower odds of HIV testing. Contrary to our hypothesis, the odds of HIV testing were higher among women, Blacks, and Latinos compared to Whites, homeless individuals, clients referred by Proposition 36 or social services, and clients who had recovery support. Results suggest that members of groups that historically have faced personal and institutional barriers to HIV testing [24–26] are receiving this service in the county system.

Although many of the factors representing the areas of the model were statistically significant, most of the ratios were small, prompting discussion of the most relevant factors observed. The models allowed us to discuss these factors by conceptual category, but it is also worth highlighting the most relevant individual factors associated with potential interventions. Among these factors was the role of age in decreased odds of receipt of HIV test results, whereas vulnerable populations like women, Blacks, and individuals with recovery support showed higher odds of HIV testing. Unlike in other studies, in Los Angeles County we found that racial and ethnic minorities are more likely than Whites to access HIV testing-related services. Although low-income racial and ethnic minorities and homeless individuals are at a disadvantage in terms of accessing HIV care and adhering to care recommendations [25, 26], there may be other factors, as suggested by our findings, that moderate these relationships. Vulnerable populations may have competing circumstances that may hinder testing, yet they may also have access to or be enrolled in programs with requirements (e.g., CalWorks welfare program, criminal justice system) that may facilitate testing and receipt of test results. These findings are also in line with studies that have shown that individuals at high risk of HIV infection as a function of their sociodemographics [43–45] and those who often engage in health and social services programs [46–48] are more likely to be tested. A possible implication of this finding is that targeting and reaching clients who may be in need of HIV testing is complex, and cannot be determined by one element of vulnerability. Multiple levels of data and knowledge, including program, geographic, and community, may be essential to developing effective interventions.

In contrast, most enabling factors were associated with higher odds of HIV testing and receipt of test results as hypothesized. As proposed, more interactions with care providers and access to employment and other health care services may increase opportunities for HIV testing and receipt of results. Findings showed that employment at admission, prior treatment episodes, residential treatment, and use of buprenorphine medication was associated with higher odds of both HIV testing and receipt of test results. It is important to note that private providers mostly prescribe buprenorphine, which may be a reflection of the socioeconomic status of clients who receive HIV testing. However, our findings are consistent with studies that have shown that access to comprehensive services and employment opportunities [44, 45, 49], as well as engagement with the health care system may present opportunities for HIV testing and awareness of HIV status [50–53]. Improving economic conditions, for example, has been associated with increased uptake of HIV testing [50, 54]. Additionally, previous encounters with treatment providers may present opportunities for education and prevention activities that are effective in increasing HIV testing [50, 51].

Need factors were also associated with higher odds of HIV testing and receipt of test results. Clients’ increased knowledge of risk factors for conditions such as HIV may increase the odds of testing and securing test results. Higher odds of HIV testing were found among clients whose primary drug of choice was alcohol or marijuana, whose method of drug use was inhalation or injection, and who had a history of mental illness or HCV or STD diagnoses. As for higher odds of receipt of test results, except for smoking, all other previously mentioned factors related to HIV testing were also positively related to receipt of test results. Overall, need and enabling factors related to clients with comorbidities or other conditions that may relate to HIV were associated with testing and receipt of results.

Although application of the Gelberg-Anderson model can include a wide array of client and provider factors that may be difficult to disentangle (e.g., use of medication, referral source, etc.) [18], it provided a critical framework to organize our findings and may inform comprehensive interventions for systems delivering care to specific vulnerable groups. Our results suggest a set of gender, race, psychosocial stressors, and drug-related conditions that may enable or inhibit HIV testing and obtaining test results. Findings from this analysis may inform the development of effective strategies to increase HIV testing and awareness of infection status among populations for whom interventions would be useful, including clients with several predisposing factors and limited enabling factors. Identifying client factors associated with HIV testing and receipt of results is also essential to improving HIV testing practices, increasing awareness of HIV status, initiating early linkage to treatment, and improving patient outcomes. Overall, our findings are important for public health because they may inform HIV testing practices and increase awareness of infection status among mostly low-income and culturally diverse clients engaged in one of the largest publicly funded addiction health services systems in the United States.

### Limitations

There are several important limitations of the study. Although members of one of the most populous and racially and ethnically diverse populations in the nation, individuals in this study represented only one county in California. Characteristics associated with HIV testing and receipt of testing results among individuals in substance use disorder treatment may differ nationally and regionally. The current analysis also focused on HIV testing prior to entering treatment. Future research should include a longer time period. Additionally, due to lack of data, we could not verify the accuracy of data on HIV testing or receipt of test results. Access to medical records would have allowed us to match administrative data with clinical records to determine whether a client was ever tested for HIV. Similarly, review of medical records may have provided information regarding whether a client was offered posttest counseling and referred to treatment or provided information on how to remain HIV negative. Last, we could not determine from the available data whether clients were offered and refused HIV testing. The data were limited regarding the availability of information about system-level factors (e.g., funding, regulation, and service infrastructure) of critical importance to delivering HIV testing and communicating results. However, these robust findings, based on 4 years of data from Los Angeles County, are important because they suggest identifiable characteristics that can be targeted to reduce the risk and spread of HIV infection among the most high-risk populations, particularly clients of addiction health services.

## Conclusion

Our findings highlight important factors that may be useful in helping substance use disorder treatment programs increase HIV testing rates, promote awareness of infection status, and prevent HIV infection and transmission. These findings are partly supported by prior studies [2, 14, 24, 26] that identified conditions associated with HIV testing in health care settings. Because client demographics, sociocultural characteristics, and provider conditions play a role in the extent to which clients receive HIV testing and obtain test results, it is critical to build on this preliminary analysis of key factors to develop longitudinal studies to assess risk and likelihood of testing. Examining these factors in one of the largest publicly funded treatment systems in the United States is of critical importance to identifying potential factors and target populations and developing strategies to increase access to and uptake of HIV testing and awareness of HIV status while decreasing the burden of HIV in populations at higher risk of contracting and spreading the disease.
